# Anlotinib-temozolomide combination achieves durable response in multimodal-resistant primary breast angiosarcoma with skull base metastasis and dural involvement: a case report

**DOI:** 10.3389/fonc.2026.1619754

**Published:** 2026-04-27

**Authors:** Shujie Song, Weiyi Chen, Xixi Li, Yongxia Zhang, Yanchun Wang

**Affiliations:** 1 Department of Medical Oncology, The Affiliated Yantai Yuhuangding Hospital of Qingdao University, Yantai, Shandong, China; 2 Department of Pathology, The Affiliated Yantai Yuhuangding Hospital of Qingdao University, Yantai, China; 3 Department of Radiology, The Affiliated Yantai Yuhuangding Hospital of Qingdao University, Yantai, China

**Keywords:** anlotinib, breast angiosarcoma, dural invasion, skull base metastasis, temozolomide

## Abstract

Primary breast angiosarcoma is an exceedingly rare malignancy with a high propensity for hematogenous metastasis, though skull base and dural involvement have never been reported. We present a 52-year-old woman diagnosed with stage IIA primary breast angiosarcoma (pT2N0M0) treated with mastectomy and adjuvant radiotherapy. Twenty-one months post-radiation therapy, she developed left orbital swelling and headache. Magnetic resonance imaging (MRI) revealed a 45×35-mm heterogeneously enhancing mass that extended from the skull base to the orbit with dural invasion, confirmed histologically as metastatic angiosarcoma. Following progression after radiotherapy and six cycles of anthracycline-based chemotherapy, salvage therapy combining anlotinib, a multi-target antiangiogenic tyrosine kinase inhibitor, and temozolomide, a blood-brain barrier-penetrant alkylating agent, was initiated. Within one week, her headache resolved, and three-month MRI demonstrated stable disease with resolution of temporalis enhancement and reduced dural thickening. The regimen was well-tolerated with no adverse events. At five-month follow-up, the patient remains progression-free. To our knowledge, this report describes the first documented case of skull base metastasis and dural involvement from primary breast angiosarcoma. The anlotinib-temozolomide combination achieved rapid symptomatic and radiographic control after multimodal therapy failure, suggesting synergistic efficacy against both angiogenesis and sanctuary-site disease. This report underscores the importance of targeting angiogenesis and leveraging central nervous system (CNS)-penetrating agents in angiosarcoma with atypical CNS dissemination, offering a novel therapeutic strategy for this aggressive malignancy.

## Introduction

Angiosarcomas are rare aggressive sarcomas of endothelial origin with a dismal prognosis (1). Representing less than 1% of all soft tissue sarcomas, they exhibit a broad clinical spectrum, arising either sporadically (primary angiosarcoma) or in association with known risk factors such as prior radiation therapy or chronic lymphedema (secondary angiosarcoma) across age groups and anatomical sites (1, 2). Regardless of etiology, angiosarcomas carry a poor prognosis, with a median overall survival of 49 months for localized disease but only 10 months once metastasis occurs (3).

Primary angiosarcoma of the breast is exceedingly rare, accounting for only 0.04% of all breast malignancies (4). This tumor is characterized by aggressive behavior, high rates of local recurrence, and a propensity for hematogenous metastasis, most commonly to the lungs, liver, or bones (4, 5). To the best of our knowledge, no case of skull base metastasis with dural involvement originating from primary breast angiosarcoma has been reported in the literature.

Herein, we present a unique case of a 52−year−old woman with primary breast angiosarcoma who developed skull base metastasis with dural invasion after multimodal therapy failure. The metastases were successfully controlled with a combination of anlotinib—a multi−target antiangiogenic tyrosine kinase inhibitor—and temozolomide, a blood–brain barrier−penetrating alkylating agent. This report highlights the potential of this regimen in managing refractory central nervous system (CNS) metastases from breast angiosarcoma and expands the known spectrum of metastatic sites for this rare malignancy.

## Histological analysis

Immunohistochemical staining was performed on formalin-fixed, paraffin-embedded tissue sections. The primary antibodies used included: CD31 (Rabbit monoclonal, Abcam, ab9498); CD34 (Mouse monoclonal, Dako, M7165); ERG (Rabbit monoclonal, Cell Marque, 760-4579); CK (AE1/AE3, Dako, M3515); Vimentin (Vim) (Mouse monoclonal, Dako, M0725); SMA (Mouse monoclonal, Dako, M0851); S-100 (Rabbit polyclonal, Dako, Z0311); ER (Rabbit monoclonal, Roche/Ventana, SP1); PR (Rabbit monoclonal, Roche/Ventana, SP2); HER2 (Mouse monoclonal, Roche/Ventana, 4B5); Ki-67 (Mouse monoclonal, Dako, M7240). Tissue sections were deparaffinized, rehydrated, and subjected to antigen retrieval by heating in citrate buffer (pH 6.0) at 95 °C for 20 minutes. Sections were then incubated with primary antibodies at the recommended dilutions for 1 hour at room temperature, followed by detection using a standard HRP-conjugated secondary antibody and DAB visualization. Hematoxylin was used for counterstaining. CD31, CD34 and ERG were used to confirm the vascular endothelial origin of the tumor cells; CK and S−100 were used to exclude epithelial and neural crest-derived tumors, respectively. Vimentin positivity supported mesenchymal origin, while SMA was assessed to rule out smooth muscle neoplasms. Given the breast origin of the primary tumor, we also evaluated HER2, estrogen receptor (ER), and progesterone receptor (PR). In addition, Ki-67 immunostaining was performed to evaluate proliferative activity.

## Case history and presentation

A 52-year-old Chinese woman with a 2-year history of a left breast mass and no family history of breast or ovarian cancer presented to an external hospital in December 2021. The patient reported no trauma, no chemical exposure, and no prior radiation to the chest wall before the detection of the breast mass. She had no significant past medical history and was not on any long-term medications. Core biopsy revealed a spindle cell tumor with vasoformative features. A left-sided mastectomy with axillary lymph node dissection (December 3, 2021) confirmed grade I angiosarcoma (pT2N0M0, Stage IIA, AJCC 8th edition). Immunohistochemistry demonstrated CD31/CD34/ERG positivity and a Ki-67 index of 15%. The patient was subsequently referred to our hospital for adjuvant radiotherapy.

**Figure 1:**
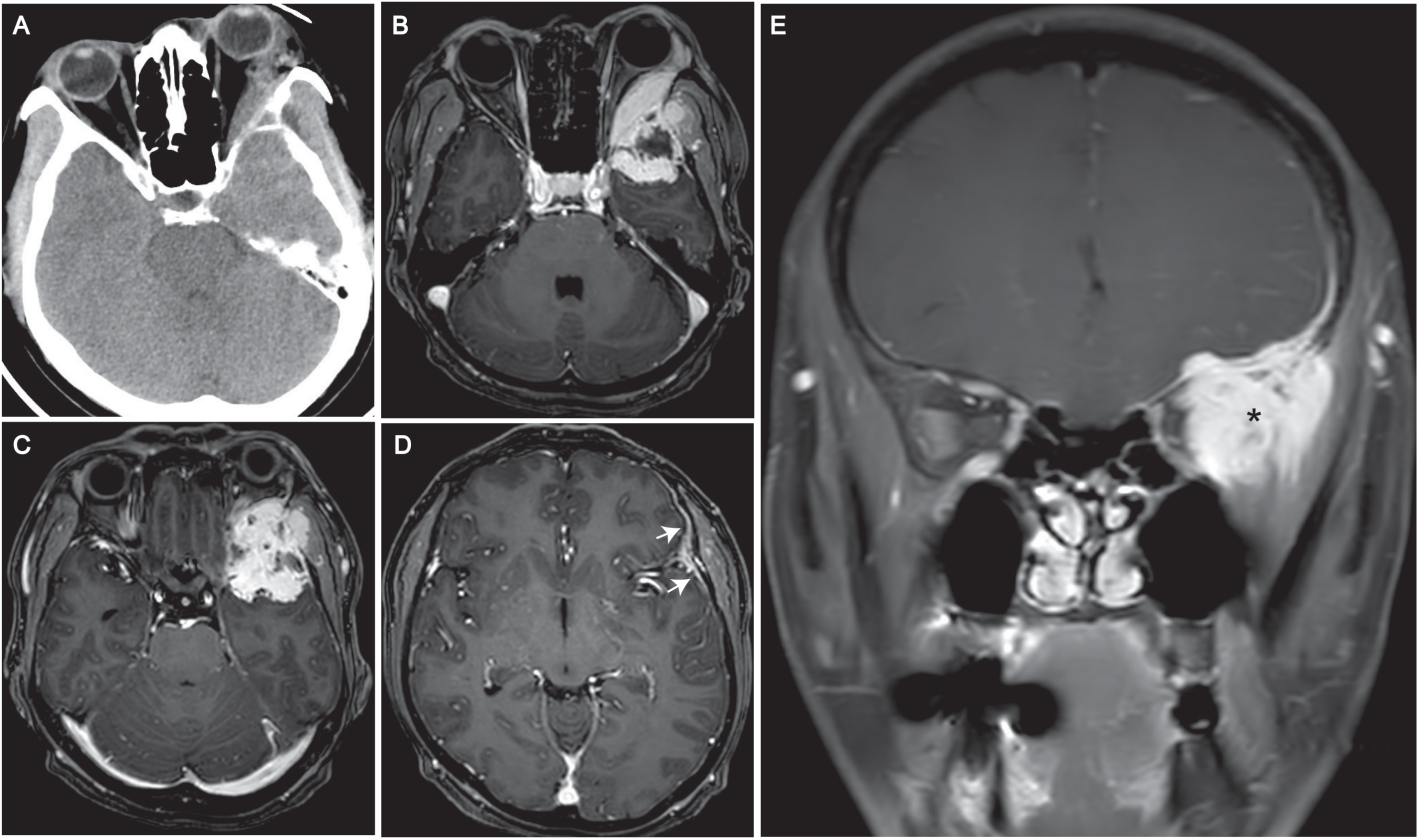
Baseline imaging at diagnosis of skull base and dural metastasis secondary to primary breast angiosarcoma. **(A)** Axial computed tomography (CT) demonstrates osteolytic destruction of the left sphenoid bone involving the skull base. **(B, C)** Axial T1-weighted contrast-enhanced magnetic resonance (MR) images reveal a heterogeneously enhancing mass centered in the left sphenoid bone. The lesion extends into the left orbital cavity with invasion of the lateral rectus muscle. Posterior extension involves the left temporalis muscle and causes mass effect on the adjacent temporal lobe parenchyma. **(D)** Axial T1-weighted contrast-enhanced MR image shows dural thickening with linear enhancement (arrows), consistent with dural metastasis. **(E)** Coronal T1-weighted contrast-enhanced MR image illustrates transcompartmental tumor growth with heterogeneous enhancement, involving the left anterior skull base, intracranial compartment and orbital cavity (asterisk).

**Figure 2:**
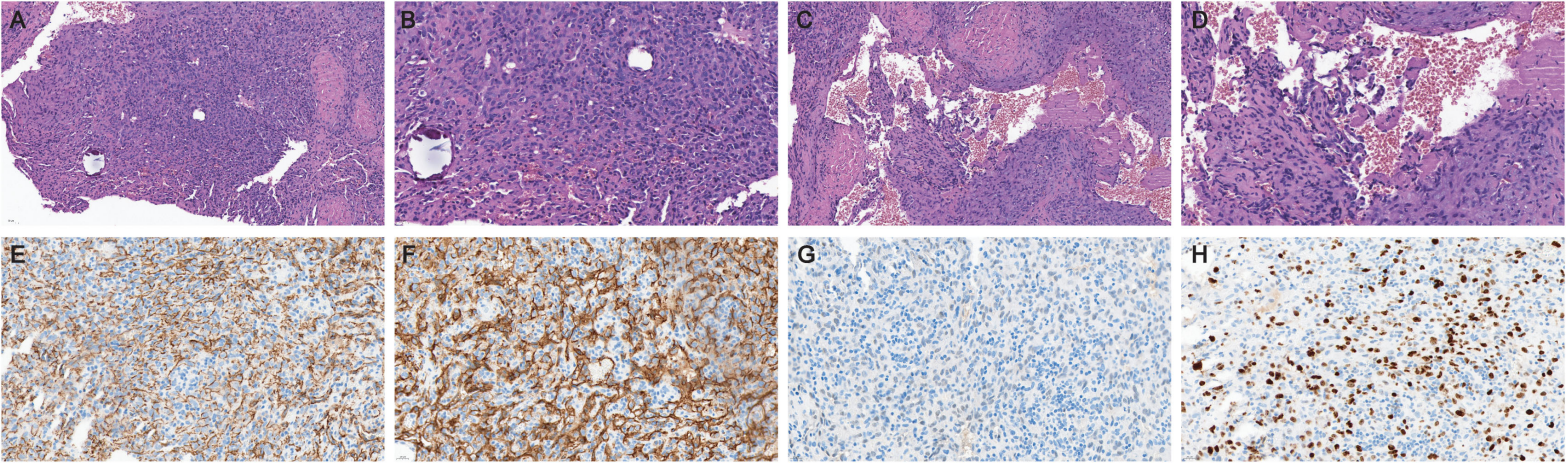
Histology of biopsy specimens of skull base metastasis. **(A)** Poorly-differentiated tumor region exhibiting a solid growth pattern with focal aberrant angiogenesis (hematoxylin and eosin [H&E], 200x). **(B)** Spindle-shaped and epithelioid tumor cells lining vascular lumens containing erythrocytes, accompanied by stromal lymphocytic infiltration (H&E, 400x). **(C)** Well-differentiated region showing irregular anastomosing vascular channels with intraluminal papillary projections (H&E, 200x). **(D)** Hypercellular vascular walls with multilayered endothelial proliferation and tufted intraluminal growth. Marked nuclear pleomorphism is evident characterized by nuclear enlargement, hyperchromasia, and irregular contours (H&E, 400x). **(E–H)** Immunohistochemical profiling of the metastatic lesion: diffuse positivity for CD34 **(E)** and CD31 **(F)**, weak ERG expression **(G)**, and an elevated proliferative index (Ki-67 30%, **(H)** (All immunohistochemical stains: 200x).

Adjuvant radiotherapy (50 Gy in 25 fractions with a 10 Gy boost) was completed in February 2022. Approximately 21 months post-radiation therapy, the patient developed left orbital swelling accompanied by mild headaches, with preserved visual acuity, no diplopia, no ptosis, no facial numbness, and no obvious cranial nerve deficits on detailed neurologic examination. Computed tomography (CT) showed an irregular mass in the skull base and the orbit cavity, with osteolytic destruction of the left sphenoid bone (**Figure 1A**). Brain magnetic resonance imaging (MRI) revealed aggressive metastatic involvement of the left skull base and dura mater (**Figures 1B–E**). A 45×35-mm heterogeneously enhancing mass was identified in the skull base and the lateral part of the orbit, centered at the sphenoid bone. Post-contrast T1-weighted sequences demonstrated marked enhancement of the mass, consistent with the hypervascular nature of angiosarcoma. The lesion extended into the left orbital cavity with invasion of the lateral rectus muscle. Posterior extension involves the left temporalis muscle and causes mass effect on the adjacent temporal lobe parenchyma. Concurrent dural metastasis was noted, characterized by dural thickening and linear enhancement. Coronal imaging further illustrates transcompartmental tumor growth with heterogeneous enhancement, involving the left anterior skull base, intracranial compartment and orbital cavity. CT scans of the chest, abdomen, and pelvis showed no evidence of other metastatic disease. On November 9, 2023, the patient underwent a core needle biopsy of the mass under general anesthesia. Significant intraoperative bleeding occurred, requiring neurosurgical intervention for hemostasis. The patient received transfusions of red blood cells, plasma, and clotting factors and was transferred to the intensive care unit (ICU) postoperatively. Histopathology confirmed skull base metastasis of breast angiosarcoma, with immunohistochemical profiling demonstrating diffuse CD31/CD34 positivity, weak ERG expression, a Ki-67 proliferative index of 30%, and negative expression of HER2, estrogen receptor (ER), and progesterone receptor (PR) (**Figures 2A–H**). Subsequently, intensity-modulated radiotherapy (IMRT) was administered to the metastases at a total dose of 59.4 Gy delivered in 27 fractions (2.2 Gy per fraction).

**Figure 3:**
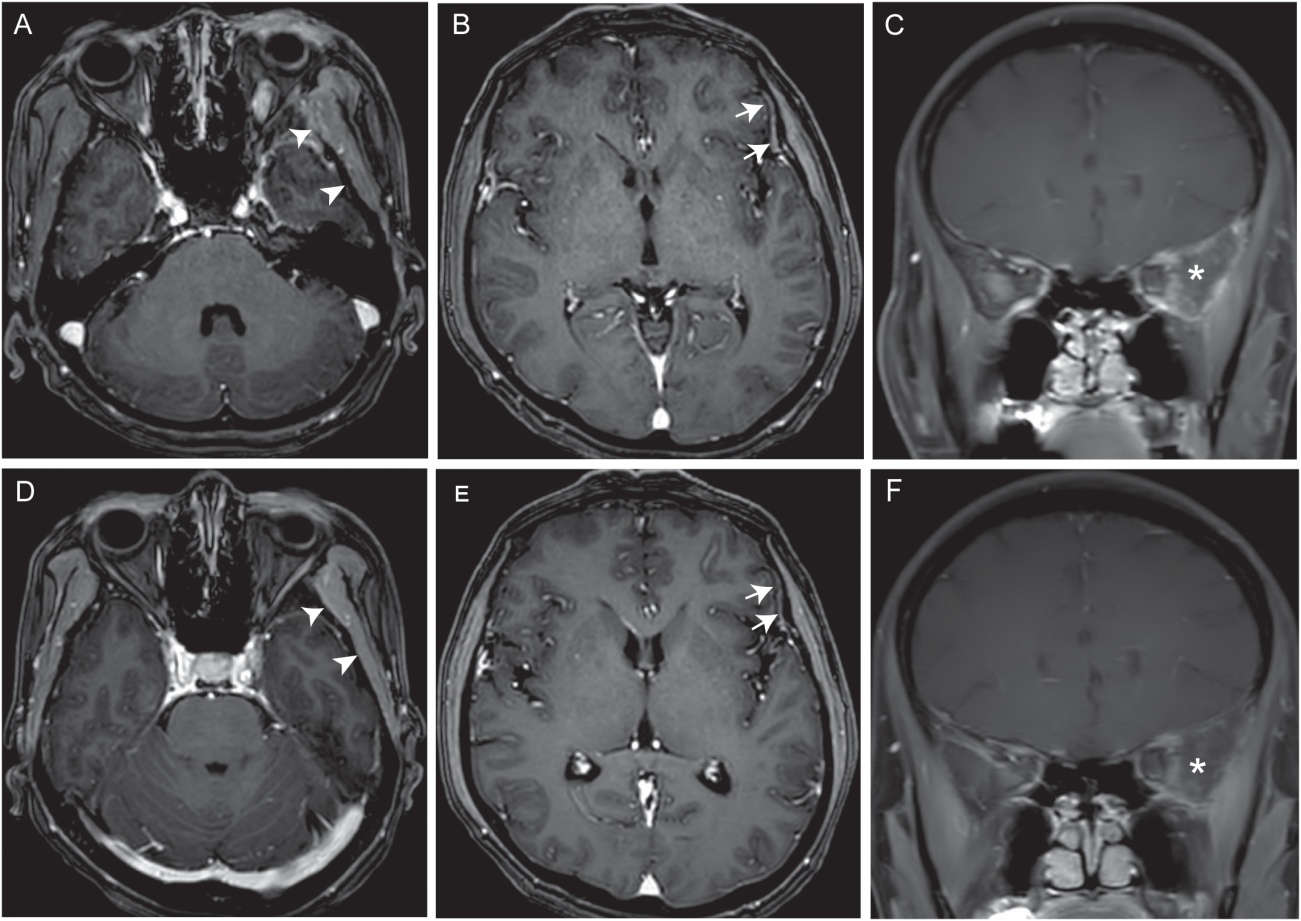
Radiologic response to combination therapy with anlotinib and temozolomide. **(A–C)** Baseline contrast-enhanced T1-weighted brain MRI before treatment initiation. Axial **(A, B)** and coronal **(C)** T1-weighted contrast-enhanced brain MRI images show abnormal enhancement of the left temporalis muscle **(A)** arrowhead and dura mater **(B)** arrows, and an enhancing mass centered on the left anterior skull base, intracranial compartment and orbital cavity **(C)** asterisk. **(D–F)** Follow-up imaging after 3 months of therapy. Corresponding axial **(D, E)** and coronal **(F)** sequences indicate complete resolution of temporalis muscle enhancement **(D)**, arrowhead, normalization of dural signal intensity **(E)**, arrows, and marked reduction in lesional enhancement **(F)**, asterisk.

One-month post-radiotherapy, follow-up brain MRI showed mild reduction in tumor size and decreased contrast enhancement, consistent with stable disease per RECIST version1.1. The patient subsequently completed six cycles of consolidation chemotherapy with epirubicin and ifosfamide, achieving a partial response after three cycles. However, disease progression was documented six months after completion of chemotherapy. Brain MRI revealed local progression, characterized by new abnormal enhancement of the left temporalis muscle, dura mater and skull base metastasis (**Figures 3A–C**).

**Figure 4:**
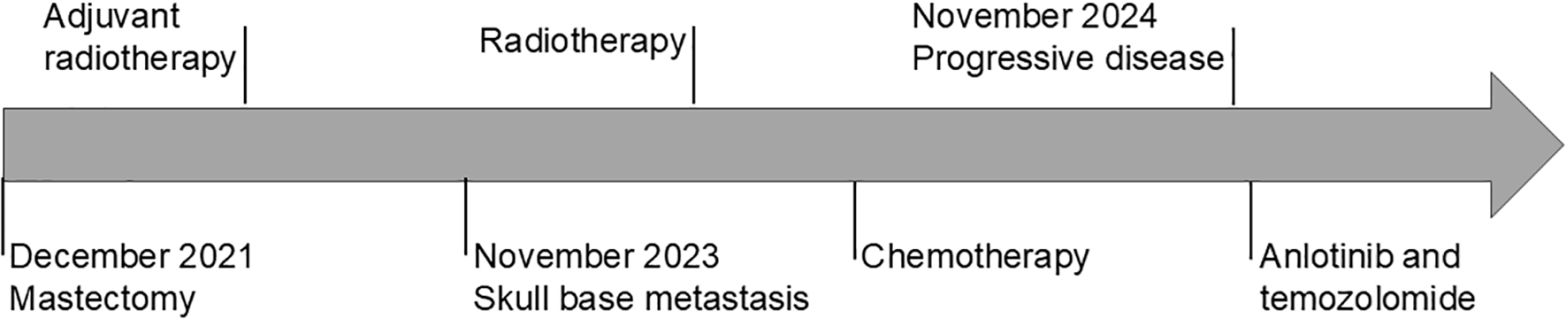
Clinical timeline of the patient’s disease course and treatment. Key events from initial diagnosis through the most recent follow-up are illustrated.

In November 2024, combination therapy was initiated with anlotinib (10 mg/day orally, days 1–14; 3-week cycles) and temozolomide (300 mg/day, days 1–5; 4-week cycles). Headache improvement was noted within one week of treatment initiation. Follow-up brain MRI at three months revealed stable disease per RECIST version1.1 criteria, characterized by no tumor enlargement, complete resolution of temporalis muscle enhancement, normalization of dural signal intensity, and significant reduction in lesional contrast uptake (**Figures 3D–F**). Systemic imaging remained negative for additional metastases. The regimen was well-tolerated with no reported adverse effects. At five-month follow-up, the patient maintains good clinical status with ongoing anlotinib-temozolomide therapy and will undergo regular surveillance to monitor long-term therapeutic efficacy. The patient’s complete clinical course is summarized in the clinical timeline (**Figure 4**). Written informed consent for publication was obtained from the patient.

## Discussion

This case represents the first documented instance of skull base metastasis arising from primary breast angiosarcoma, a malignancy already notorious for its rarity and aggressive behavior. Angiosarcomas, particularly those originating in the breast, pose significant therapeutic challenges due to their propensity for hematogenous spread, resistance to conventional therapies, and poor prognosis in metastatic settings (1, 6, 7). The development of skull base metastasis and dural invasion in this patient underscores the tumor’s capacity for atypical dissemination, further complicating management in an already dismal prognostic landscape (median survival of 10 months for metastatic disease) (7, 8).

Primary breast angiosarcoma accounts for only 0.04% of breast malignancies, with metastatic spread typically involving the lungs, liver, or bones (4, 5). CNS metastases from breast angiosarcoma are exceedingly rare, with only isolated case reports in the literature. Rozen et al. reported a 53-year-old woman with breast angiosarcoma who developed an isolated pituitary metastasis 9 months after diagnosis (9). Despite surgical debulking of the pituitary lesion, the patient developed progressive respiratory failure secondary to multiple pulmonary emboli, further complicated by a malignant pleural effusion. Active treatment was withdrawn and she was transferred to a palliative care institution. This highlights the aggressive nature of CNS involvement and limited therapeutic options. Our patient received radiotherapy and systemic therapy, which may have contributed to better local control. However, we also acknowledge that differences in tumor behavior and molecular profiles likely played a role. To date, skull base metastasis with dural invasion, has not been previously reported. This case expands the spectrum of metastatic sites associated with angiosarcoma and highlights the importance of vigilant neurological monitoring in patients with advanced disease. The observed Ki-67 index escalation from 15% in the primary tumor to 30% in the metastatic lesion suggests heightened proliferative activity, potentially contributing to the aggressive recurrence.

The failure of standard therapies—surgery, radiotherapy, and anthracycline-based chemotherapy—suggests resistance of this patient’s angiosarcomas to conventional regimens. The subsequent stabilization achieved with anlotinib, a multi-target TKI of VEGF/PDGF receptors that was approved in China for the treatment of advanced soft tissue sarcoma after failure of prior systemic therapies, and temozolomide, an alkylating agent with CNS penetration, offers critical mechanistic insights. Angiosarcomas are highly vascular tumors driven by aberrant angiogenesis, making antiangiogenic strategies a rational therapeutic avenue (10, 11). However, clinical experience with antiangiogenic agents in angiosarcoma has been disappointing overall. Phase II studies of sorafenib in advanced angiosarcoma have shown only modest activity, with median progression−free survival of approximately 4 months and overall survival around 8 months (12). Similarly, combinations such as paclitaxel plus bevacizumab have yielded objective responses in a subset of patients but have not clearly translated into a substantial survival advantage compared with chemotherapy alone (13).

Anlotinib’s broader target profile, including VEGFR, FGFR, and PDGFR, may provide more comprehensive blockade of pro−angiogenic signaling, and this could contribute to reduced tumor vascularization, as suggested by the decreased contrast enhancement on MRI (14). At the same time, the blood–brain barrier (BBB) remains a significant obstacle for most systemic therapies, including taxanes and gemcitabine (15), whereas temozolomide is well established to cross the BBB and is a cornerstone of glioblastoma treatment (16). Temozolomide’s role in penetrating the blood-brain barrier may have augmented efficacy against dural metastasis, a sanctuary site often shielded from systemic therapies (16). The rationale for selecting the anlotinib-temozolomide combination is further supported by emerging evidence in CNS tumors. A phase II study of anlotinib plus temozolomide in recurrent glioblastoma reported an objective response rate of 81.0% and a 6−month progression−free survival rate of 61.9%, with manageable toxicity, and subsequent real−world data have shown median progression−free and overall survival of 7.8 and 17.9 months, respectively, in recurrent glioma (17, 18). These data provide a translational rationale for applying this combination to CNS metastases from angiosarcoma, which shares the highly vascularized phenotype with glioblastoma. These findings provide a translational rationale for combining anlotinib with temozolomide in skull base/dural metastases from angiosarcoma, which share a highly vascular phenotype with glioblastoma. The stable disease observed in our patient contrasts with the rapid progression reported after radiotherapy and chemotherapy in metastatic angiosarcoma, particularly with CNS involvement, and suggests that this strategy may merit further investigation in selected patients (7, 9, 19).

As a single-case report, generalizability is limited, and the 5-month follow-up precludes conclusions regarding long-term outcomes. Larger cohorts and prolonged observation are needed to validate this regimen’s efficacy and tolerability. Additionally, due to the small amount of biopsy tissue obtained (complicated by intraoperative bleeding) and the limited residual sample (prior extensive diagnostic immunohistochemistry), we were unable to perform molecular profiling. After all, molecular profiling of the tumor could help elucidate predictive biomarkers for antiangiogenic response, optimizing patient selection.

## Conclusion

This case illustrates the potential of combining an antiangiogenic agent with CNS-penetrating chemotherapy in refractory angiosarcoma, particularly for atypical metastases. The stabilization achieved with anlotinib and temozolomide underscores the importance of targeting tumor vasculature while addressing the unique challenges of CNS involvement. Further research into targeted therapies and their combinations is imperative to improve outcomes for this rare, aggressive malignancy.

## Data Availability

The raw data supporting the conclusions of this case report are not publicly available due to ethical and privacy restrictions. De-identified data may be requested from the corresponding author upon reasonable request, subject to the patient’s consent.
